# Human immunodeficiency virus infection and inflammatory arthritis: A review of clinical and imaging features

**DOI:** 10.4102/sajr.v21i2.1261

**Published:** 2017-11-14

**Authors:** Farhana E. Suleman, Mahmood M.T.M. Ally

**Affiliations:** 1 Department of Radiology, University of Pretoria, South Africa; 2 Department of Internal Medicine, University of Pretoria, South Africa

## Abstract

The reported prevalence of articular manifestations of human immunodeficiency virus (HIV) varies, but with sub-Saharan Africa accounting for almost 70% of the people living with HIV, this results in a considerable burden of disease in the region. The spectrum of clinical presentation described, includes articular pain syndrome, HIV-associated arthropathy and seronegative spondyloarthropathies, among others. This brief review serves to create awareness of the clinical and imaging presentation of this spectrum of disease as there is significant morbidity associated with these conditions if treatment is delayed.

## Introduction

The global human immunodeficiency virus (HIV) pandemic has impacted on the burden of disease seen in clinical practice, and this includes the spectrum of inflammatory arthritis. While the reported prevalence varies, patients with HIV are proven to be at higher risk of developing a large spectrum of these pathologies.^1^ A brief review of the clinical and radiological findings is presented in this article.

## Arthralgia

Arthralgia is the most common but non-specific articular complaint of HIV-positive patients. It tends to occur in the acute phase of the infection and is the result of direct infection of the joint by the virus. The frequently affected joints are the knees, shoulders and elbows. It may manifest as a mono-, oligo- or polyarticular disease. It can be transient or intermittent and may require analgesia.^1,2,3^ Imaging features are normal.

## Painful articular syndrome

Painful articular syndrome is described less commonly than arthralgia but is characterised by acute severe debilitating pain of short duration lasting less than 24 h. It has been reported to occur in the late stages of HIV infection^2^ and commonly affects the knees, shoulders and elbows.^3^ Clinically there are no signs of synovitis.^4^ Imaging features may be normal, but sometimes a joint effusion is noted. Peri-articular osteopaenia may or may not be present.^5^

## HIV-associated arthritis

**FIGURE 1:**
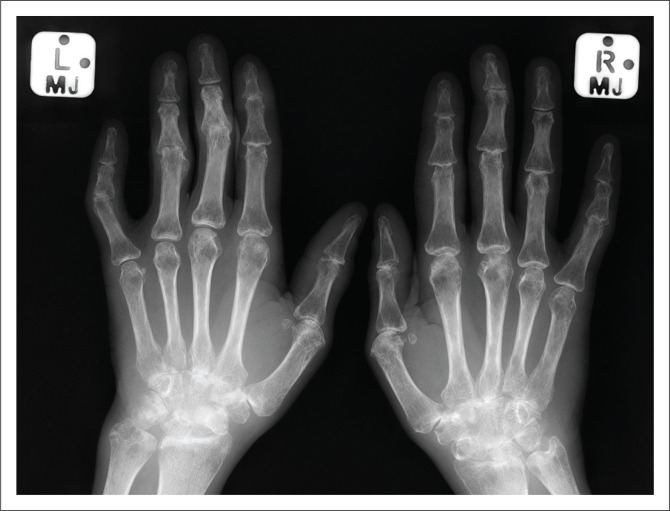
Frontal radiographs of both hands in an HIV-positive patient presenting with polyarthritis. RF was negative. Radiographs demonstrate bilateral severe peri-articular osteopaenia, severe joint space narrowing involving almost all joints and subluxation at the 3rd, 4th and 5th proximal interphalangeal joints.

**FIGURE 2:**
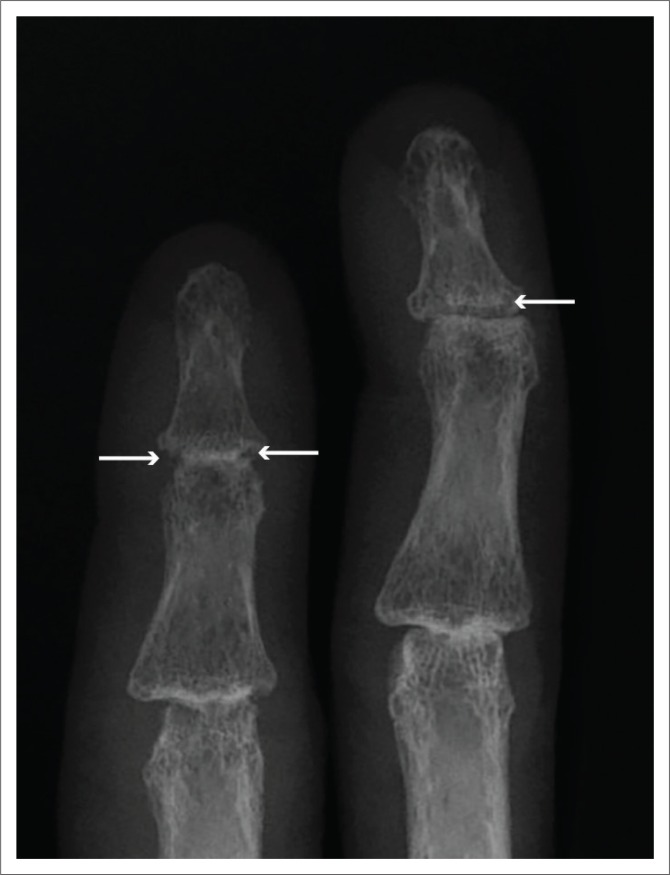
Magnified view of the 2nd and 3rd distal interphalangeal joints of the same patient discussed in Figure 1 demonstrates early erosions and irregularity of the distal phalanges (arrows).

**FIGURE 3:**
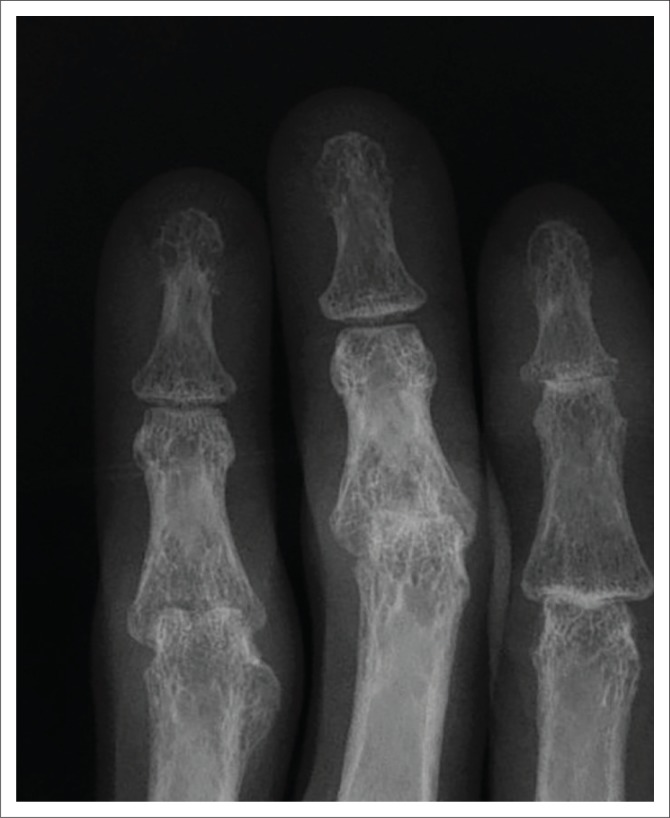
Magnified view of the phalanges of the left 2nd, 3rd and 4th phalanges of the left hand of the patient discussed in Figure 1, demonstrating new bone formation of the proximal phalanx of the 4th finger and more clearly demonstrates the subluxations at the 3rd and 4th proximal interphalangeal joints.

**FIGURE 4:**
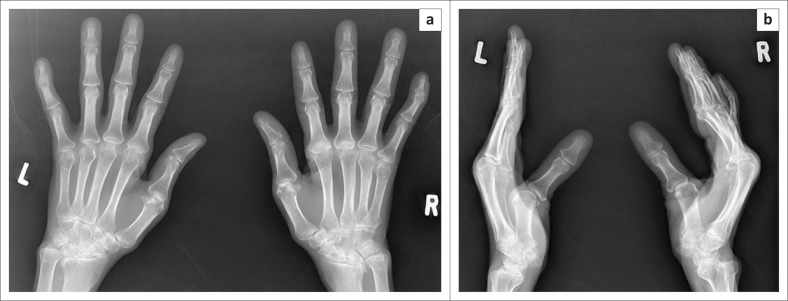
(a) Frontal and (b) lateral radiographs of both hands of an HIV-positive patient presenting clinically with reducible deformities and arthralgia. Rheumatoid factor and anti-nuclear factor were negative. Lateral radiographs show subluxations at the metacarpophalangeal joints bilaterally as well as the distal interphalangeal joints of both 5th fingers. The right hand is more affected than the left, with the left hand demonstrating more reducible deformities on the frontal radiograph compared to the right hand.

**FIGURE 5:**
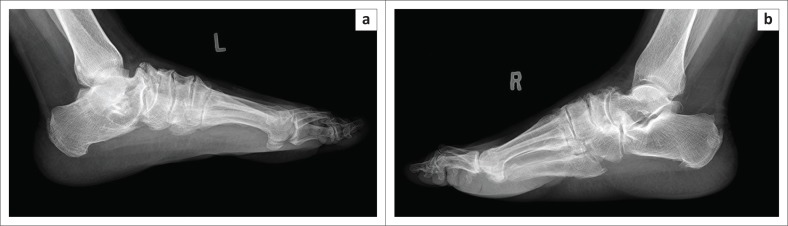
Lateral radiographs (a and b) of both feet in an HIV-positive patient. The radiograph demonstrates periostitis and enthesitis affecting the right foot more than the left foot.

**FIGURE 6:**
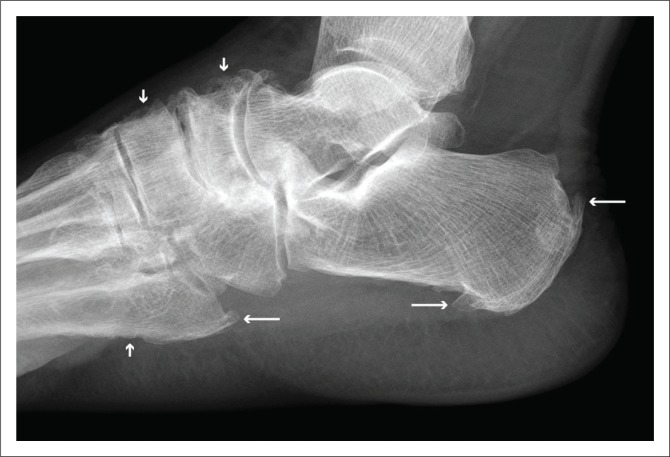
Magnified view of the proximal metatarsals and tarsal bones of the right foot clearly demonstrating periostitis (short arrows) and enthesitis (long arrows).

HIV-associated arthritis has been described more commonly in patients from sub-Saharan Africa.^4^ Serological tests such as rheumatoid factor (RF) and anti-nuclear factor antibodies associated with inflammatory arthritides are negative, as is the HLA B27 genotype. They also do not fulfil the criteria for the spondyloarthropathies.^6,7^ It may occur at any stage of HIV infection and most often presents as an asymmetric oligoarthritis more commonly in men.^7^ The lower limbs, especially the knees, are most commonly affected,^1,2,3,4,5,6,7^ and it is described as self-limiting, lasting less than 6 weeks.^2,5,7^ A symmetrical polyarthritis pattern resembling rheumatoid arthritis (RA) may also be seen.^5,6,7^ Deformities similar to RA are described although the onset is more acute and less erosive (Figures 1–3). A Jaccoud arthropathy may also form part of this spectrum (Figure 4).^2,7^ A small number of patients may progress to a chronic destructive arthritis and imaging findings similar to RA may be noted including peri-articular osteopaenia, erosions and joint space narrowing. Periostitis and proliferative new bone formation helps to distinguish the condition from RA (Figures 5 and 6).^5,7^

## Reactive arthritis (Reiter’s Syndrome)

A typical presentation of seronegative peripheral oligoarthritis predominantly affecting the lower limbs as seen in the HIV-negative population is described in most of the literature. This is accompanied by marked enthesitis evidenced by Archilles tendinitis, plantar fasciitis and tenderness at sites of entheses.^2,3,4,5,6,7^ Involvement of the spine and sacroiliac joints is less commonly seen.^6^ Mucocutaneous involvement is common (circinate balanitis and keratoderma blenorrhagicum).^2,3,4,5,6,7^ Extensive psorasiform skin manifestations have been described, that may cause confusion with a diagnosis of psoriasis.^2,3,4,7^ A history of preceding gastrointestinal or genitourinary tract infection is also common.^7^

Imaging changes are described predominantly in the joints of the lower limbs, including small joints of the foot with changes of erosion and adjacent new bone proliferation noted.^5^ These changes tend to be asymmetrical and may lead to progressive deformity and severe arthritis of the larger joints.^2^ Erosions and spur formation are typically noted at entheseal sites.^5^

## Psoriatic arthritis

An increased incidence as well as a more severe and persistent form of psoriasis has been described in HIV-positive patients.^7^ The associated arthritis has also been described as more destructive and erosive, as well as more refractory to treatment.^7^ It tends to occur late in the course of HIV infection and is usually a sign of subsequent opportunistic infections.^6,7^

Patients may present with an asymmetrical oligoarthritis or polyarthritis predominantly affecting the lower limbs.^2,6,7^ Involvement of the spine and sacroiliac joints is less common. Patients may rapidly progress to a deforming and debilitating course. A symmetrical polyarthritis may also occur that may mimic RA.^5,7^

Imaging features may include erosions, joint space narrowing, ankylosis, joint effusions, soft tissue swelling and deformities. Soft tissue swelling is noted at entheseal sites such as at the insertion of the Archilles tendon and digital swelling from dactylitis. The presence of periostitis, new bone formation and distal interphalangeal joint involvement helps to distinguish the condition from RA.^5^

## Undifferentiated spondyloarthropathy

Patients who do not exhibit a full spectrum of clinical manifestations that allow them to be classified as ankylosing spondylitis, psoriatic arthritis or reactive arthritis are labelled as undifferentiated spondyloarthropathy.^2,4,6,7^ The most common manifestation is enthesitis, adjacent osteitis, synovitis and spur formation with involvement of the knee, a common imaging finding.^2,7^ The epidemic of HIV infection has seen a marked increase in the incidence of this condition in sub-Saharan Africa.^2^

## Rheumatoid arthritis

There is still ongoing debate about the effects of HIV on RA.^2^ Some reports in the literature suggest that RA patients may go into remission with HIV infection, but there are also reports that suggest that the disease may flare or arise *de novo* during the immune reconstitution inflammatory syndrome.^6^ HIV-associated polyarthritis may also mimic RA clinically and radiographically, creating difficulties with diagnosis. Biomarkers of RA have also been detected in low titres in patients who are HIV-positive.^7^

## Conclusion

A large burden of HIV infection resides in sub-Saharan Africa. This has resulted in a changing spectrum of inflammatory arthritis seen in an HLA B27 negative population, where it was previously, relatively uncommon. An awareness of the clinical and radiological manifestations of the variety of diseases is important for early diagnosis and management to prevent disability and further morbidity of a range of conditions that have the potential to cause severe incapacity.
